# Blastomycosis in Minnesota, USA, 1999–2018

**DOI:** 10.3201/eid2605.191074

**Published:** 2020-05

**Authors:** Malia Ireland, Carrie Klumb, Kirk Smith, Joni Scheftel

**Affiliations:** Minnesota Department of Health, St. Paul, Minnesota, USA

**Keywords:** Blastomyces, blastomycosis, fungi, epidemiology, inhalation exposure, treatment outcome, antifungal agents, itraconazole, lung diseases, pneumonia, risk factors, delayed diagnosis, endemic diseases, incidence, sex ratio, geography, travel, public health, Minnesota, United States

## Abstract

Blastomycosis is a systemic disease caused by *Blastomyces* spp. fungi. To determine its epidemiology in blastomycosis-endemic Minnesota, USA, we evaluated all cases reported to public health officials during 1999–2018. We focused on time to diagnosis, exposure activities, and exposure location. A total of 671 cases and a median of 34 cases/year were reported. Median time to diagnosis was 31 days; 61% of patients were not tested for blastomycosis until they were hospitalized. The case-fatality rate was 10%, and patients who died were 5.3 times more likely to have a concurrent medical condition. Outdoor activities and soil exposure were reported by many patients, but no specific activity or exposure was common to most. Almost one third of patients were probably exposed in geographic areas other than their home county. Providers should consider alternative etiologies for patients with pneumonia not responding to antibacterial treatment, and public health officials should increase awareness in blastomycosis-endemic areas.

## Abstract 2

Providers should consider a blastomycosis diagnosis for pneumonia patients who have been in disease-endemic areas.

Blastomycosis is a systemic disease caused by thermally dimorphic *Blastomyces* spp. fungi found in soil. Infection with *B. dermatitidis* or *B. gilchristii* occurs primarily by inhalation of conidia and most often causes pneumonia, although direct inoculation of soft tissue can occur (*1*). Infections can disseminate hematogenously, most commonly to skin, bone, and the central nervous system (*2*). Case-fatality rates range from 6% to 22% (*3*–*5*). Most patients are male (60%–75%) (*3*–*8*) and middle-aged (median age 41–44 years) (*3*,*5*,*7*,*8*). Diagnosis is often delayed because community-acquired bacterial pneumonia has a similar presentation (*9*,*10*) and index of suspicion for blastomycosis is low among healthcare providers (*1*,*11*). The standard diagnostic method is isolation and identification of *Blastomyces* spp. in culture from clinical specimens, but also used are histopathology, cytopathology, antigen testing, and antibody testing (*1*).

In North America, blastomycosis occurs primarily in areas surrounding the Great Lakes, the Mississippi and Ohio River valleys, and the St. Lawrence River, which include many US states and Canada provinces (*1*,*2*). Recent phylogenetic studies and ecologic niche modeling reports have increased our knowledge of the distribution and ecology of *Blastomyces* spp. (*12*–*15*). However, the difficulty of isolating the organism directly from environmental samples limits our ability to determine its true endemic ranges (*12*). Case series and outbreak reports have provided insight into the ecology of *Blastomyces* spp. and possible risk factors for human infection (*16*–*19*). Outbreaks have been associated with outdoor recreation (*17*,*20*–*22*) and with construction, excavation, or local environmental sources such as yard waste compost (*18*). Incidence or mortality rates are increased among black (*3*,*4*,*23*), Asian (*24*), American Indian/Alaska Native (*23*), and Aboriginal Canadian persons (*5*). Risk factors for sporadic cases are less well documented; a retrospective case–control study did not find associations with classic outbreak exposures (*4*).

To better describe the epidemiology of blastomycosis in Minnesota, an endemic area, we evaluated all cases reported to public health officials during 1999–2018. We also examined delayed recognition and diagnosis of the disease.

## Methods

Blastomycosis has been reportable to the Minnesota Department of Health (MDH) since 1985. Beginning in 1999, MDH routinely collected information on demographics, illness history, diagnostic test results, treatment, outcomes, and any exposures by using a standardized case report form. Data collection evolved over time; during 1999–2015, case report forms were completed by providers or their staff, and during 2016–2018, MDH staff abstracted medical records and completed case report forms. During the entire study period, a confirmed case of blastomycosis was defined as illness in a Minnesota resident with any of the following: a positive *Blastomyces* culture, *Blastomyces* organisms visualized in tissue or body fluid, or a positive *Blastomyces* antigen test result and compatible clinical illness (e.g., cough, fever, abnormal pulmonary imaging, or skin lesions). Cases were classified as pulmonary only, nonpulmonary (localized disease outside the pulmonary system with no clinical pulmonary illness), or disseminated (disease in both the pulmonary system and at least 1 other system/site). We collected illness onset date, date of first visit to a healthcare provider, and date of the first test for blastomycosis regardless of test result. To assess diagnostic delays, we defined the patient interval as the time between illness onset and first visit to healthcare and the provider interval as the time between first healthcare visit and sample collection date for the first blastomycosis test (which indicates that a blastomycosis diagnosis was under consideration). Total time to diagnosis was defined as the time from illness onset to the first test for blastomycosis. We used the date of first test regardless of result to evaluate the time until healthcare providers considered a systemic mycotic infection. Doing so eliminated the variability in growth rate of *Blastomyces* cultures.

We attempted to interview all patients or next of kin regarding patients’ illness and exposure history during the 3 months before illness onset, including home and neighborhood environment, occupation, outdoor activities and travel, concurrent medical conditions, immunosuppressive medications, smoking history, and family members or pets with a blastomycosis diagnosis. Underlying conditions included diabetes mellitus, chronic lung disease (e.g., chronic obstructive pulmonary disease, asthma), chronic liver disease (e.g., cirrhosis, hepatitis), and other chronic illnesses (e.g., HIV infection/AIDS, sarcoidosis, heart disease, kidney disease). Immunosuppressive medications included corticosteroids, tumor necrosis factor–α blockers, chemotherapy, or posttransplant medications. Patients were also asked about any information missing from case report forms regarding demographics, symptoms, and prescribed antibacterial and antifungal drugs. On the basis of exposure information obtained during interviews, we assigned the most likely location of *Blastomyces* exposure for each patient, either a specific Minnesota county or an out-of-state location. This subjective assessment considered incubation period, travel, and activities.

We included in our analysis confirmed cases with a positive specimen collection date of 1999 through 2018. We did not include patients with positive antigen test results but no compatible illness, positive serologic antibody tests only, or other fungal infections.

We calculated incidence by race by using the number of cases and race population in Minnesota for each year (*25*) and then averaged the yearly incidence rates. We calculated incidence by county by using the average population of each county for the entire period and the average number of cases in each county. We classified counties with an incidence rate of >3 cases/100,000 population as highly blastomycosis-endemic counties, based on a natural break in the distribution of incidence by county.

We analyzed data by using SAS 9.2 statistical software (https://www.sas.com) and conducted univariate analysis by using χ^2^, Fisher exact, Student *t*, Wilcoxon rank-sum, and Kruskal-Wallis tests. To control for race and sex in analyses of outcome and concurrent conditions, we used multivariate logistic regression. We considered 2-sided p-values of <0.05 to be significant.

## Results

### Demographics

**Figure 1:**
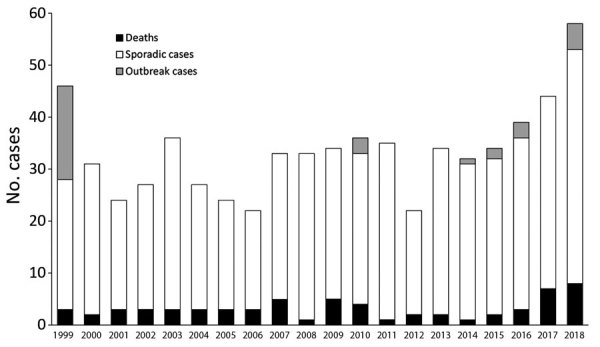
Blastomycosis cases per year, Minnesota, USA, 1999 –2018. No deaths occurred in patients with outbreak cases.

During the 20-year study period, 671 confirmed cases of blastomycosis were reported in Minnesota; the median number of cases per year was 34 (range 22–58) (Figure 1). A total of 32 (5%) cases were part of outbreaks with patient exposure in Minnesota or Wisconsin, including a large 1999 outbreak in St. Louis County, Minnesota, which involved humans and dogs and was associated with wet weather and an excavation site for a new neighborhood. Except for 1999, more cases were reported during 2016–2018 than during previous years.

**Figure 2:**
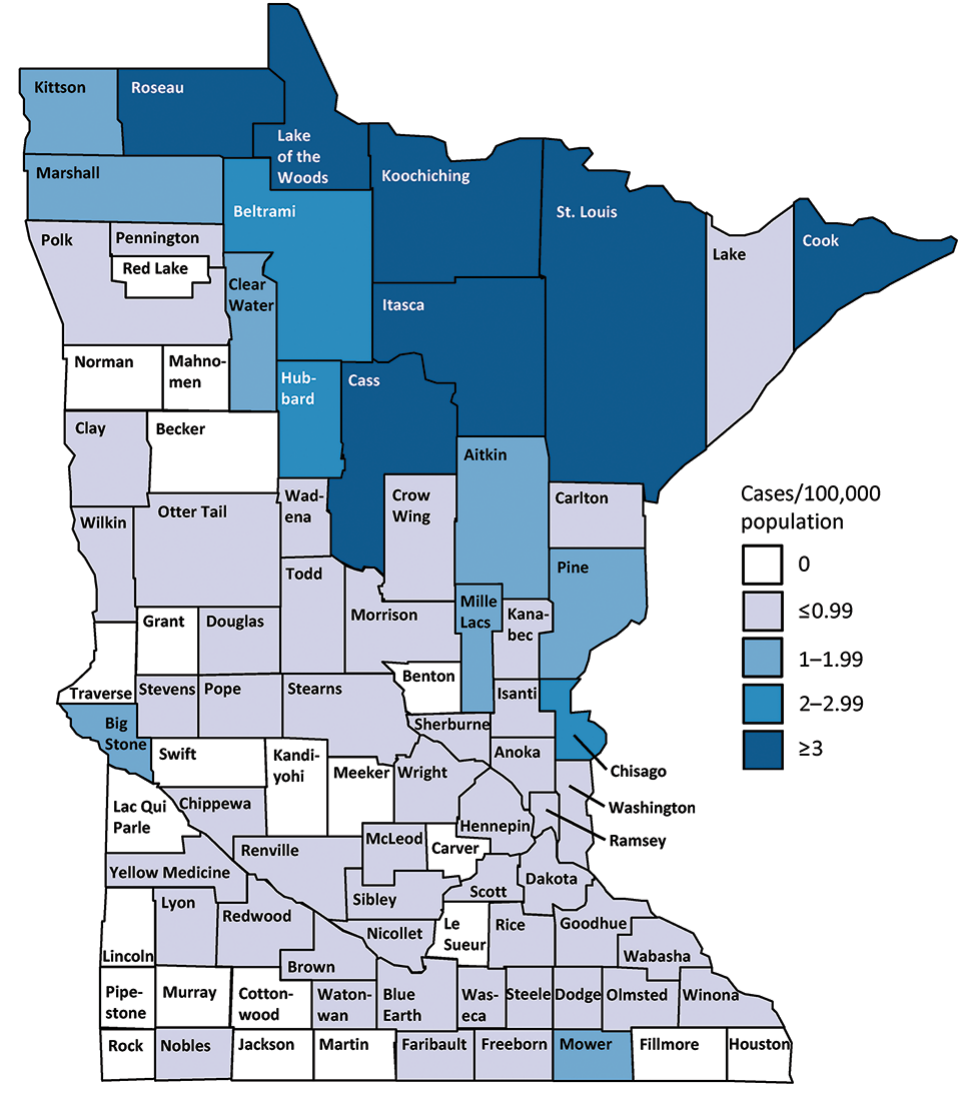
Average annual incidence of blastomycosis by county, Minnesota, USA, 1999–2018.

**Figure 3:**
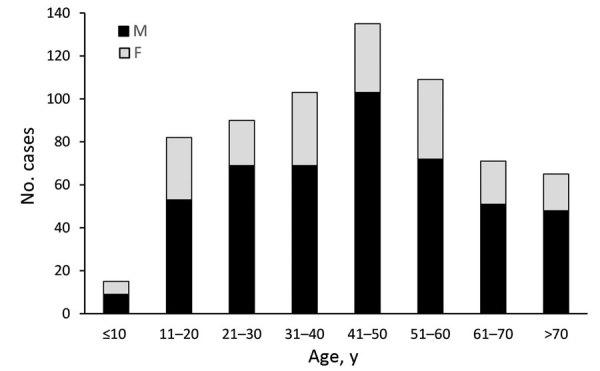
Age and sex distribution of 670 patients with blastomycosis, Minnesota, USA, 1999–2018.

The statewide average annual incidence was 0.64 cases/100,000 population. Average annual incidence ranged from 0 to 7.6 cases/100,000 for individual counties (Figure 2). The median patient age was 44 years (range 3–93 years), and 474 (71%) patients were male (Table 1; Figure 3). The average annual incidence was highest for American Indian/Alaska Natives (2.7/100,000 population), followed by white (0.53/100,000), Asian/Pacific Islander (0.51/100,000), and black (0.48/100,000) persons.

**Table 1:** Blastomycosis patient demographics and health-related data, by outcome, Minnesota, USA, 1999–2018*

Category	All patients, n = 671	Patients with nonfatal cases, n = 602	Patients with fatal cases, n = 64	OR (95% CI)†	p value†
Mean age ± SD, y	44 ± 19.2	42 ± 18.5	57 ± 20.7		p<0.001‡
Sex				NS	
M	474 (71)	426 (71)	45 (70)		
F	197 (29)	176 (29)	19 (30)		
Race				NS	
White	490 (83)	446 (84)	44 (75)		
American Indian/Alaska Native	40 (7)	32 (6)	7 (12)		
Black	31 (5)	27 (5)	3 (5)		
Asian/Pacific Islander	23 (4)	19 (4)	4 (7)		
Other/Mixed race	4 (1)	4 (1)	0		
Ethnicity				NS	
Non-Hispanic	439 (96)	398 (96)	41 (95)		
Hispanic	18 (4)	16 (4)	2 (5)		
Location of disease				NS	
Pulmonary only	469 (72)	425 (72)	43 (69)		
Disseminated	137 (21)	117 (20)	19 (31)		
Nonpulmonary	46 (7)	46 (8)	0		
Concurrent conditions					
Any	195 (35)	157 (30)	36 (71)	5.3 (2.7–10.5)	p<0.001
Chronic illnesses	109 (22)	87 (19)	20 (57)	5.5 (2.6–11.6)	p<0.001
Diabetes mellitus	81 (17)	68 (16)	11 (42)	3.6 (1.5–8.7)	p = 0.005
Chronic lung disease	15 (4)	11 (3)	4 (21)	10.2 (2.7–38.6)	p<0.001
Immunocompromise	46 (11)	34 (8)	12 (44)	8.6 (3.4–21.8)	p<0.001
Steroid use	15 (4)	8 (2)	7 (32)	21.1 (6.3–70.8)	p<0.001
Rheumatoid arthritis treatment	13 (3)	9 (2)	4 (21)	10.6 (2.3–48.8)	p = 0.002
Cancer	24 (6)	19 (5)	5 (25)	7.7 (2.4–25.5)	p<0.001

*Values are no. (%) except as indicated. Denominators may not be same across categories because not all data were available for all patients. OR, odds ratio; NS, not significant. †ORs and p values calculated by using univariable logistic regression, except for concurrent conditions analyses, which were controlled for race and sex. ‡p value calculated by using Student *t *test.

### Clinical Characteristics

Reported symptoms included cough (83%), fatigue (79%), fever (69%), weight loss (62%), night sweats (61%), poor appetite (57%), chills (57%), joint pain (30%), back pain (28%), and skin lesions (25%). A total of 456/663 (69%) patients were hospitalized for a median of 8 (range 1–197) days (hospitalization data were not available for 8 patients). Most (72%) infections involved only the pulmonary system, 21% of infections were disseminated, and 7% were nonpulmonary localized infections (Table 1). The most common site was skin or soft tissue for disseminated infections (108 cases, 79%) and nonpulmonary infections (38 cases, 83%), followed by bones or joints (22 [16%] disseminated cases, 6 [13%] nonpulmonary cases) and the central nervous system (13 [9%] disseminated cases, 2 [4%] nonpulmonary cases). For 47% of patients, >3 courses of antibacterial drugs were prescribed before blastomycosis was diagnosed.

### Diagnostic Methods

The most commonly reported diagnostic test used was culture; 557/617 (90%) positive results were reported. Positive cytopathology results were reported for 250/539 (46%) patients and positive histopathology results for 83/467 (18%). Samples obtained by bronchoalveolar lavage or tracheal swab were the most common sources for culture (269/567 [47%] patients) and cytopathology (123/273 [45%] patients). Antigen testing became available in 2003 but was not widely used until 2008. Of the 401 patients from 2008–2018, a positive urine or serum antigen test was included in the diagnostic testing for 167 (42%). Use of antigen tests to evaluate treatment efficacy was not tracked.

### Time to Diagnosis

**Figure 4:**
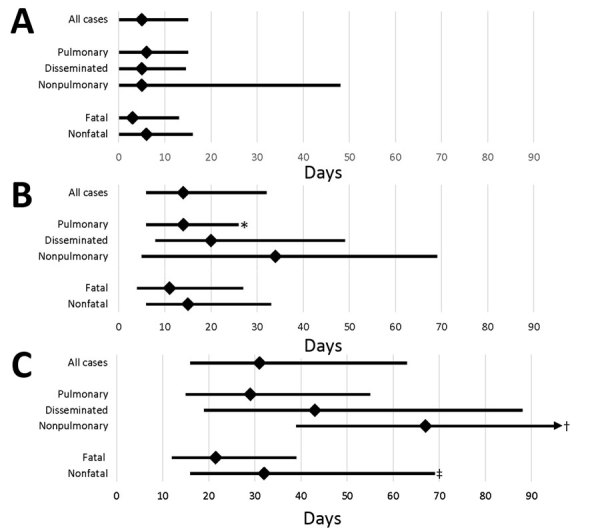
Treatment delay for blastomycosis patients, by category of case, Minnesota, USA, 1999–2018. A) Patient interval (time of illness onset to first healthcare visit); B) provider interval (time of first visit to first blastomycosis test); and C) total time to diagnosis (time of illness onset to first test). Median (diamonds) and interquartile ranges (error bars) are shown. p values were calculated by using the Kruskal-Wallis and Wilcoxon rank tests and are indicated when significantly different from others in category. *p<0.001; †3rd quartile = 150; p = 0.009; ‡p = 0.001.

Among all patients, the median total time to diagnosis was 31 (range 0–1,130) days, interquartile range [IQR] 16–64 days) (Figure 4). The median patient interval (time from illness onset to first visit) was 5 (range 0–409, IQR 0–15) days. The median provider interval (time from first visit to first blastomycosis test) was 14 (range 0–517, IQR, 6–32) days (Figure 4). Provider interval was >30 days for 27% of patients. The median total time to diagnosis for patients with nonpulmonary disease was 67 (IQR 39–150) days. For 61% of patients, blastomycosis testing was not performed until they were admitted to the hospital. The median time to diagnosis was 40.5 (IQR 22–58) days for Asian/Pacific Islander patients and 34 (IQR 12–60) days for American Indian/Alaska Native patients compared with 31 (IQR 16–66) days for white patients. However, these differences were not statistically significant.

The overall time to diagnosis was the same in both highly blastomycosis-endemic and less blastomycosis-endemic counties. Hospitalization was more likely for patients living in less blastomycosis-endemic counties (72%) than for patients living in highly blastomycosis-endemic counties (64%) (odds ratio [OR] 1.4, 95% CI 1.02–1.99; p = 0.036).

### Treatment

Medications used for blastomycosis treatment were not reported for all case-patients. Among those for whom they were reported, 462 (84%) case-patients received itraconazole, 145 (26%) amphotericin B, 37 (7%) voriconazole, and 28 (5%) other or unknown medications.

### Outcomes

The overall case-fatality rate was 10% (yearly range 3%–16%). Although fatality rates were higher among persons in some racial groups (18% for American Indian/Alaska Natives, 17% for Asian/Pacific Islanders), no statistically significant differences were observed. Patients who died were significantly older than patients who survived; mean difference was 15 years (Table 1). No patients with nonpulmonary infections died. Among patients who did die, the median time to diagnosis was significantly shorter than for those who survived (Figure 4).

### Concurrent Conditions

Concurrent medical conditions or an immunocompromised status resulting from illness or medication were reported by 195 (35%) patients (Table 1). Patients who were hospitalized were twice as likely as those not hospitalized to have a concurrent condition (OR 2.12, 95% CI 1.4–3.2; p<0.001). Patients who died were 5.3 times more likely to have a concurrent condition than were patients who survived (Table 1). The most common concurrent condition was diabetes. Current smoking was reported by 109 (20%) patients, and any history of smoking was reported by 194 (39%).

### Exposures

Interviews were conducted with 541 (81%) of 671 patients or their next of kin (Table 2). In the 3 months before illness onset, 317 (59%) of 539 patients participated in >1 outdoor activity including hunting, fishing, swimming, boating, camping, hiking, or biking. Of these, the most commonly reported were boating (40%), fishing (30%), and hiking (28%). At least 1 specific soil exposure was reported by 375 (78%) of 480 patients; soil exposures included gardening, clearing or cutting wood, gathering wild plants, using an all-terrain vehicle (ATV), or being near excavation. Occupational exposure to soil, wooded, or boggy areas was reported by 97 (21%) of 468 patients. None of these activities or other exposures were reported by 31 (6%) patients. Owning a dog was reported by 283 (53%) of 539 patients; of those, 29 (10%) reported owning a dog that had ever had blastomycosis. Having a family member who had ever had blastomycosis was reported by 22 (4%) of the 541 patients. A significantly higher proportion of male than female patients reported hunting, fishing, and using an ATV (Table 2), but we found no significant differences by sex for hiking, camping, gardening, nearby excavation, beaver dam exposure, or owning a dog. We also found no significant differences in exposures by sex among children <16 years of age.

**Table 2:** Exposures and associations of interviewed blastomycosis patients, by sex, Minnesota, USA, 1999–2018*

Exposures and associations	All cases, no. (%), n = 541	Male patients, no. (%), n = 383	Female patients, no. (%), n = 158	OR (95% CI)†	p value†
Outdoor activity	317 (59)	245 (64)	72 (46)	2.1 (1.5–3.1),	p<0.001
Hunting	84 (15)	80 (21)	4 (2)	10.5 (3.8–29.2)	p<0.001‡
Fishing	161 (30)	134 (35)	27 (17)	2.7 (1.7–4.3)	p<0.001
Boating	99 (40)	78 (44)	21 (30)	1.8 (1.0–3.2)	p = 0.049
Swimming	59 (27)	47 (29)	12 (21)	NS	
Camping	87 (16)	66 (17)	21 (13)	NS	
Hiking	152 (28)	111 (29)	41 (26)	NS	
Specific soil exposure	375 (78)	276 (80)	99 (72)	1.6 (1.0–2.5)	p = 0.050
ATV use	86 (19)	74 (23)	12 (10)	2.8 (1.5–5.4)	p = 0.001
Wood clearing/cutting at home	193 (35)	165 (43)	28 (17)	3.6 (2.3–5.7)	p<0.001
Garden	148 (34)	101 (34)	47 (36)	NS	
Nearby excavation	172 (32)	124 (33)	48 (30)	NS	
Visiting a cabin	177 (33)	131 (35)	46 (29)	NS	
Nearby beaver dams	86 (16)	67 (18)	19 (12)	NS	
Working in woods§	97 (21)	88 (26)	9 (7)	4.9 (2.4–10.1)	p<0.001
Family member(s) had blastomycosis	22 (4)	11 (3)	11 (7)	0.39 (0.16–0.93)	p = 0.029
Owning a dog	283 (53)	197 (52)	86 (54)	NS	
Owning a dog that had blastomycosis	29 (10)	19 (9)	10 (11)	NS	

*Denominators may not be same across categories because not all data were available for all patients. ATV, all-terrain vehicle; OR, odds ratio; NS, not significant. †ORs and p values calculated by using χ^2^ test, except where indicated. ‡p value calculated by using Fisher exact test. §Part-time or full-time employment in wooded areas (e.g., construction, landscaping, forestry).

Patients with prior medical conditions were less likely (47%) than previously healthy patients (64%) to report participation in outdoor activities (OR 0.49, 95% CI 0.33–0.71; p<0.001). The individual activities of fishing, camping, hiking, and swimming were reported significantly less often by patients with concurrent conditions (data not shown).

**Figure 5:**
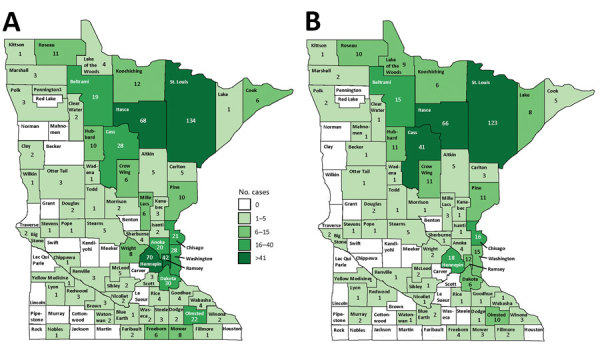
Blastomycosis cases, by county of residence (A; n = 670) and probable county of exposure (B; n = 463), Minnesota, USA, 1999–2018.

A total of 340 (51%) of 671 patients were most likely exposed to *Blastomyces* in their county of residence; 195 (29%) were exposed outside their county of residence, either in other Minnesota counties or other states (Figure 5). These locations included the highly blastomycosis-endemic northern Minnesota counties of St. Louis (27 patients), Cass (24), and Itasca (10); Wisconsin (52); and Canada (10). The most probable location of exposure was unknown for 136 patients (20%) because of multiple possible locations (21 patients [3%]) or because no interview could be conducted (115 patients [17%]).

## Discussion

The epidemiology and clinical courses of blastomycosis cases in Minnesota are similar to those in other disease-endemic regions. The case-fatality rate, sex ratio, age distribution, and reported symptoms are consistent with those reported from other disease-endemic areas (*3*,*5*,*7*,*8*). Although case counts and fatality rates were higher in the most recent 2 years, we found no overall trends by year, race, or sex. The typical blastomycosis patient in Minnesota is a 45-year-old white man who spends time outdoors. However, this report highlights underrecognized features of blastomycosis epidemiology, particularly patients who do not fit the typical presentation or demographic.

Although the blastomycosis incidence rate was highest among American Indian/Alaska Natives, the incidence difference between that group and white, Asian, and black persons was not statistically significant. However, in Minnesota, the population of American Indian/Alaska Natives is much smaller than that of other races, which, combined with other factors such as prevalence rates for concurrent conditions or geographic location of residence, may influence incidence rates in the American Indian/Alaska Native population. For example, a larger proportion of American Indian/Alaska Native blastomycosis patients (70%) than patients of other races (38%) live in highly blastomycosis-endemic counties (OR 3.9, 95% CI 1.9–7.8; p<0.001). Genetics may also play a role. Others have discussed increased susceptibility to disease or severe disease after *Blastomyces* infection for Asian, black, and American Indian/Alaska Native persons (*3*,*4*,*19*,*23*,*24*,*26*). Our data show higher case-fatality rates for Asians and American Indians/Alaska Natives; however, the difference was not significant, even after controlling for sex and underlying conditions. Another study evaluating mortality rates found that the likelihood of dying from blastomycosis-related complications was higher for American Indian/Alaska Native and black patients than for white patients (*23*). Variation in blastomycosis incidence and outcomes by race warrants further exploration (*26*).

Hospitalization and mortality rates were higher among patients with underlying conditions or immunocompromised status, as previously reported (*5*,*7*,*27*). Those patients reported outdoor and soil exposures less frequently than did previously healthy patients, which could lead a clinician to discount blastomycosis as a diagnostic possibility. Because the odds of death are dramatically higher for patients with an underlying condition than for previously healthy patients, earlier consideration of alternative pneumonia etiologies (beyond antimicrobial drug–resistant bacterial infection) for those patients is warranted.

Diagnosis of blastomycosis is often delayed. For half of the patients in this study, >1 full month elapsed between illness onset and the patient’s first test that could diagnose blastomycosis. Diagnosis took even longer for those with nonpulmonary infections. Provider interval accounted for a larger proportion of this time than did patient interval. Medical record abstraction provided ample anecdotal evidence that patients visited healthcare providers numerous times before their first blastomycosis test. These delays have many possible consequences, including unnecessary antibacterial drug use and higher hospitalization rates. Being hospitalized seemed to be a key factor in being tested for blastomycosis; 60% of patients were not tested until hospital admission. Earlier testing may have prevented some of these admissions. Relatively few patients underwent urine or serum antigen testing, which, despite cross-reactivity with other fungal pathogens, may have provided guidance toward a general diagnosis of fungal disease and appropriate treatment.

Time to diagnosis should logically be shorter for patients with skin lesions because visible lesions might trigger sampling or consideration of blastomycosis in a patient with concurrent pneumonia. This result was found for 123 Mississippi patients, for whom clinicians correctly diagnosed 64% of blastomycosis cases for patients with skin lesions on their initial visit but only 18% of blastomycosis cases for all patients (*28*). However, in our study, we compared patients with skin lesions with patients without skin lesions and found that provider delay and total time to diagnosis were significantly longer for those with skin lesions than without skin lesions (data not shown).

Time to diagnosis was also significantly shorter for patients who died than for those who survived. Both patient interval and provider interval were shorter for patients who died, probably because their blastomycosis was more severe from the onset, which may have resulted in earlier presentation to healthcare and more aggressive initial diagnostics. However, patients who died were not tested for a median of 11 days after their first healthcare visit, and perhaps some might not have died had a diagnosis been reached sooner.

We anticipated that time to diagnosis would be shorter for patients residing in highly blastomycosis-endemic counties because local providers would be more familiar with the disease and would order testing earlier. Although the median time to diagnosis was the same in highly and less disease-endemic counties, patients living in highly disease-endemic counties were 50% less likely to be hospitalized. This finding may indicate that where providers were more familiar with blastomycosis, they more frequently ordered testing before hospitalization was required.

Although incidence by county of residence provides a measure of disease frequency, 29% of patients were probably exposed outside their home county. Mapping of case totals for county of exposure compared with county of residence illustrates that many patients live in more populated, less blastomycosis-endemic counties, such as Hennepin and Ramsey (i.e., the Minneapolis–St. Paul metropolitan area) but are exposed in highly blastomycosis-endemic northern counties (e.g., Cass, Itasca). By tracking suspected exposure locations, we can more accurately distinguish highly blastomycosis-endemic areas. Further refinement of such areas and future exposure location data will provide more information about the ecologic niche of blastomycosis and help focus awareness campaigns among healthcare providers, residents, and visitors. Many of the highly blastomycosis-endemic counties attract seasonal residents and tourists in summer, underscoring the value of travel histories for patients with infectious disease and provider familiarity with geographic areas where risk for *Blastomyces* exposure is greater.

As previously reported, blastomycosis cases were skewed heavily by patient sex. This finding is often attributed to a perceived higher likelihood of male patients having engaged in recreational and occupational outdoor activities that increase risk for exposure to *Blastomyces* (*1*,*5*). In Minnesota, 2 other outdoor-associated diseases that occur in similar locations, Lyme disease and anaplasmosis, also occur more often in men, but the male:female ratio (60:40) is less dramatic (*29*). Male blastomycosis patients reported participation in some outdoor activities at significantly higher proportions than did female patients. However, even when evaluating an exposure that should not intuitively differ by sex, such as nearby excavation, 72% of patients were male. Other factors may explain differences by sex, such as hormonal effects, as have been proposed for other diseases (*30*–*32*).

A previous retrospective case–control study did not find any association between infection and factors typically associated with blastomycosis in outbreaks, such as proximity to water, recreational activities, or soil-related activities (*4*). A prospective case–control study would help determine whether those who acquire blastomycosis participate in these exposure activities at higher rates. Most patients in this study reported >1 activity typically considered a risk factor for blastomycosis, but no activities were common to all or most patients. In addition, this study does not enable us to determine which exposures present the highest risk because background exposure rates for Minnesota residents are not readily available.

Study limitations include those characteristic of surveillance data. Because the surveillance system is passive, undercounting is possible if cases were not reported. Race and ethnicity were not always documented in medical records. Data collection methods changed in 2016 when medical record abstraction was added. Back and bone pain were added to case report forms in 2001; joint pain, boating, and ATV use were added in 2010. Data regarding the number of healthcare visits were not consistently available. We used the date of first test for blastomycosis as the endpoint for determining time to diagnosis. However, negative test results are not routinely reported. Although we made every effort to collect this information from medical record abstraction or providers, some could have been missed.

In conclusion, to reduce the time to diagnosis and case-fatality rates for patients with blastomycosis, providers should consider alternative etiologies for patients with pneumonia that is unresponsive to antibacterial drugs. Complete travel and exposure histories should be obtained, and antigen testing should be considered as a screening test. Blastomycosis should be considered an emerging risk for immunocompromised or chronically ill patients in disease-endemic regions, even for those who do not report classic exposures. Public health officials should work to increase awareness among persons who live and visit blastomycosis-endemic areas so they can advocate for themselves.
